# Adherence to 7-Day Primaquine Treatment for the Radical Cure of *P. vivax* in the Peruvian Amazon

**DOI:** 10.4269/ajtmh.2010.09-0521

**Published:** 2010-06

**Authors:** Koen Peeters Grietens, Veronica Soto, Annette Erhart, Joan Muela Ribera, Elizabeth Toomer, Alex Tenorio, Tanilu Grande Montalvo, Hugo Rodriguez, Alejandro Llanos Cuentas, Umberto D'Alessandro, Dionicia Gamboa

**Affiliations:** Department of Parasitology, Institute of Tropical Medicine, Antwerp, Belgium; Instituto de Medicina Tropical Alexander von Humboldt, Universidad Peruana Cayetano Heredia, Lima, Peru; Partners for Applied Social Sciences, PASS International, Tessenderlo, Belgium; Organismo Andino de Salud, Convenio Hipolito Unanue, Iquitos, Peru

## Abstract

Despite being free of charge, treatment adherence to 7-day primaquine for the radical cure of *Plasmodium vivax* was estimated at 62.2% among patients along the Iquitos-Nauta road in the Peruvian Amazon. The principal reason for non-adherence was the perceived adverse effects related to local humoral illness conceptions that hold that malaria produces a hot state of body, which is further aggravated by the characteristically hot medical treatment. Notably, patients were willing to adhere to the first 3 days of treatment during which symptoms are most apparent and include the characteristic chills. Nevertheless, as symptoms abate, the perceived aggravating characteristics of the medication outweigh the perceived advantages of treatment adherence. Improving community awareness about the role of primaquine to prevent further malaria transmission and fostering a realistic system of direct observed treatment intake, organized at community level, can be expected to improve adherence to the radical cure of *P. vivax* in this area.

## Introduction

Treatment adherence to primaquine (PQ) for the radical cure of *Plasmodium vivax* infection is a major public-health concern in the Peruvian Amazon because of the predominance of this species in the region.^1^ Although rarely fatal, the persistence of *P. vivax* in the liver can induce relapses for years after the initial infection, making adherence particularly relevant to eliminate the latent hypnozoite reservoir after symptoms abate.^2^ In 2001, adherence problems to PQ led the Peruvian National Malaria Control Program to shorten the length of the treatment from a 14-day to a 7-day PQ course and to increase the daily dose from 0.25 to 0.5 mg/kg/day. Nevertheless, despite the simpler therapeutic protocol, *P. vivax* malaria incidence in the Peruvian Amazon region remained high with recurrent parasitemia after treatment in a high proportion of cases (T. Grande Montalvo and V. Soto, personal communication). Adherence, therefore, remains a key factor for effective malaria control, because it is directly related to treatment failure and the possible spread of resistant malaria strains. Furthermore, when patients do not access the right treatment, this results in poor health outcomes, lowering life quality and increasing medical costs.^3^,^4^

Little is known, however, about patients' motives for adherence or non-adherence in non-trial settings.^5^ The objectives of the present study were to evaluate patient adherence to the 7-day PQ course and provide additional qualitative data on factors influencing the adherence process in local communities of the Peruvian Amazon.

## Methods

### Study site.

**Figure 1.**
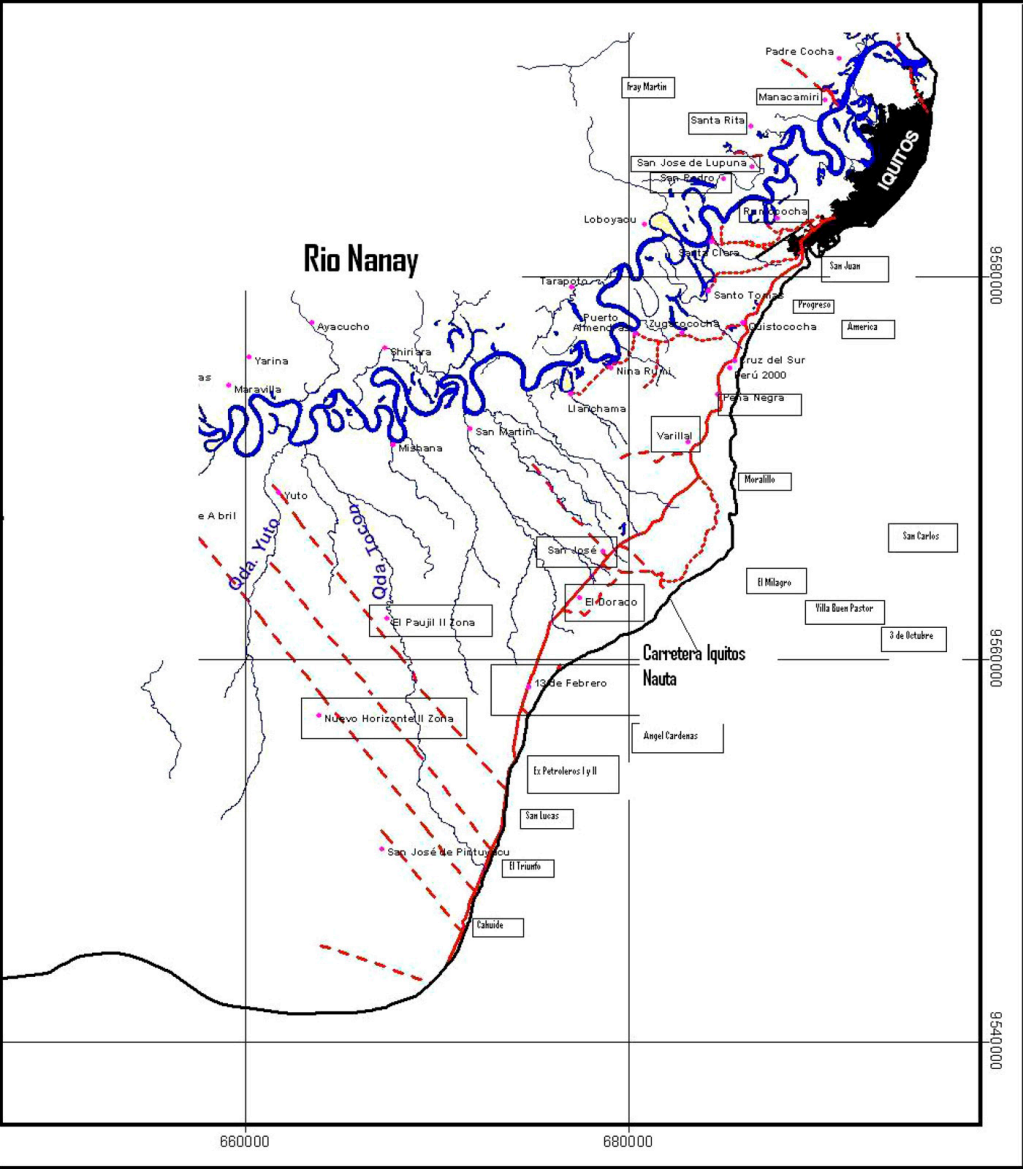
Map of the study area. This figure appears in color at www.ajtmh.org.

The study was conducted in the catchment area of the Paujil and Cahuide Health Centers (HC) in the district of San Juan situated along the road connecting Iquitos city to the small port city of Nauta (Figure 1), in the department of Loreto belonging to the Peruvian Amazon. Malaria distribution in Loreto is highly heterogeneous and contains foci of high incidence, such as the district of San Juan, which reported in 2007 the highest number of cases in the region (total of 4,075 for a population of 108,353).^1^ Malaria transmission is perennial with a peak during the rainy season between November and May. All age groups are at risk, although adults are more so than children, and most infections are symptomatic.^6^–^8^ The main vector is *Anopheles darlingi*, a highly anthropophilic species that recently (1990s) spread to this region because of new settlements and increasing forest exploitation.^9^,^10^

All communities are situated in the vicinity of the Itaya and Nanai Rivers and are within a 15-km range of the HC, and most are directly accessible by road.^11^ Nevertheless, accessibility problems are common during the rainy season and persist year round for communities that can only be reached by boat because of a lack of transportation and high fuel prices.

### Study population.

The population in the study area was estimated at 5,239 inhabitants in 2007, and it mainly consists of mestizos, which refers to all Peruvians that cannot be clearly identified as belonging to any ethnic minority population. Local subsistence strategies include slash and burn agriculture, fishing, hunting, and small-scale carbon production. Occasionally, people also engage in fish farming, logging, small commercial activities, and salaried employment as grounds' keepers or cultivators for institutions, farms, and enterprises belonging to wealthier Iquitos residents.

Traditionally, the local population uses non-impregnated bed nets made of white woven cotton or tocuyo.^12^ Long-lasting insecticidal nets were distributed with the Global Fund's support just before the start of the present study in 2007.^13^ Anti-malaria treatment is provided free of charge after microscopy diagnosis at the Paujil and Cahuide HCs. Treatment of *P. vivax* consists of 3 days of chloroquine (CQ; total = 25 mg/kg) and 7 days of PQ (0.5 mg/kg/day), and it is administered without prior screening for G6PD deficiency, which is not considered prevalent in the area, although no formal reports have been published.^14^ According to official guidelines, *P. vivax* patients are expected to report daily to the HC (except on Sundays) to receive their treatment doses, which are supervised by the health staff (Sunday doses are given 1 day in advance). However, in practice, patients are only asked to return to the HC two times: at the end of the CQ treatment to collect the remaining doses of PQ and at the end of the PQ treatment for a final blood smear.

Most local communities have a trained volunteer community health worker called *Promotor de Salud* able to take blood smears. After microscopic diagnosis at the HC, the health promoter is expected to directly observe the intake of treatment for those unable to go to the HC.

### Research strategy.

The present research was ancillary to a larger ongoing cohort study on *P. vivax* morbidity after radical cure treatment, which was carried out in the framework of an institutional collaboration between the Institute of Tropical Medicine (ITM) Antwerp-Belgium and the ITM Alexander von Humboldt (ITM-AvH) Lima-Peru.^15^ The present targeted ethnography was designed to get a quantitative estimation of adherence to the current 7-day CQ-PQ regimen of microscopically confirmed *P. vivax* malaria cases and to provide qualitative background information on treatment adherence and related decision-making factors in the local context. Considering the inherent reporting bias in response rates to adherence-related questions, methodological triangulation (the use of various methods and techniques to refine and test particular interpretations and hypotheses) was used, combining qualitative data obtained during field work and quantitative data both from patients' medical records at the HCs and from an additional survey administered to a random sample of previously treated vivax patients in their respective communities. Treatment adherence was defined as the proportion of patients that had actually completed the 7-day treatment as prescribed. Health-system performance and quality-of-case management factors were not included.

### Qualitative data collection.

During field work, the following research techniques were combined. Participant observation, including reiterated informal interviews with key and general informants, was carried out in 10 communities: El Dorado, 13 de Febrero, Nuevo Horizonte, Ex-Petroleros, 12 de Abril, Cahuide, Villa Buen Pastor, Paujil, 24 de Junio, and El Triunfo. Informal conversations were not recorded. Notes were kept of the most relevant observations and conversations. Interviews were recorded and transcribed. When recording was not considered appropriate because of the sensitivity of the subject discussed or the informality of the conversation, notes were taken immediately after the interview. The interviews were held in the selected above-mentioned locations, representing a sufficient number of respondents to get a coherent picture of the local context and social setting.

### Sampling.

Sampling for all informal and formal interviews was purposive.^16^–^19^ Informants were selected according to relevant variables such as gender, age, subsistence strategy, locality, adherence, etc. to allow for maximum variation in the sample. Sampling techniques included respondent-driven or snowball sampling to improve data reliability.^20^,^21^

### Quantitative data collection.

Two sources of quantitative data were combined to assess treatment adherence.

#### Medical records from HCs.

All *P. vivax* patients identified at the Paujil and Cahuide HCs between January 2005 and July 2007 were anonymously recorded (using only code numbers) with the total treatment dose delivered by the HC. Additionally, vivax cases belonging to the Paujil and Cahuide HCs catchment area that had reported to neighboring HCs or the Iquitos Hospital were included in the database. For each patient, the total dose delivered at the beginning and after the completion of the CQ treatment was recorded in the database. Because these figures only related to the treatment provided by health services but not to treatment intake, a random sample of the above-mentioned patients was interviewed on the actual number of doses taken.

#### Patient-adherence survey.

A sample of 185 vivax malaria patients was randomly selected from the HC database, and to minimize recall bias, the selection was limited to patients identified within the year before the study (i.e., July 2006 to July 2007). Patients were visited at home by the study team, and after oral informed consent, a structured, closed questionnaire was administered (for children < 18 years old, the questionnaire was administered to parents/guardians). The survey examined delay before consultation at the HC, productivity time loss because of the malaria episode, adherence to current radical treatment, reasons to interrupt treatment, and the alteration or combination of treatment with traditional remedies and home treatment.

### Data analysis.

#### Qualitative data.

Qualitative data were entered and analyzed using N/Vivo Qualitative Analysis software (QSR International Pty Ltd., Cardigan, UK). The analysis was a flexible and iterative process; preliminary data obtained from different techniques were entered and analyzed to generate temporary results. Further research was then conducted to confirm or refute these temporary results until saturation was reached and data could be theoretically supported.

#### Quantitative data.

All quantitative data were entered, cleaned, and analyzed in Epi Info 6.04 (Centers for Disease Control, Atlanta, GA; World Health Organization, Geneva, Switzerland). Summary statistics were computed using means or medians as appropriate for continuous variables and proportions for categorical variables. The main outcome variable was treatment adherence. Secondary outcome variables were perceived causes of malaria, perceived treatment effects, and reported timing of perceived treatment effects.

### Ethical clearance.

The study was approved by the Ethical Committee of the University of Antwerp, Belgium and the Ethical Review Committee of the Universidad Peruana Cayetano Heredia, Lima, Peru. During field work, all interviewers followed the Code of Ethics of the American Anthropological Association (AAA).^22^ As proposed by the AAA, all interviewees were informed before the start of the interview about project goals, the topic and type of questions, their right to refuse being interviewed, interrupt the conversation at any time, and withdraw any given information during or after the interview, and the intended use of the results for scientific publications and reports to health authorities. Oral consent was preferred, because the interviewees were not put at any risk of being harmed physically or psychologically and the act of signing one's name when providing information during interviews could be a potential reason for mistrust.^23^

## Results

### Socio-demographic characteristics of *P. vivax* malaria patients.

A total of 1,279 confirmed *P. vivax* patients (presence/history of fever and presence of *P. vivax* asexual forms in the blood smears) were identified at the Paujil and Cahuide HCs between January 2005 and July 2007 (for patients with multiple vivax episodes, only the last episode was recorded). Among these patients, 1,072 (83.8%) had complete information on the number of CQ and PQ doses given. The majority of patients were male (61.9%; 664/1,072), and the median age was 21 years (range = 1–81 years). No geographic clustering was found. Patients' subsistence strategies were mostly centered around slash and burn agriculture in combination with various other economic activities such as fishing, hunting, coal production, fish farming, logging, and gathering of certain palm leaves.

### Perceived cause of malaria infection.

The perceived cause of the last *P. vivax* episode was asked to the 185 randomly selected patients in their respective communities (Table 1). Malaria infection was mostly related to (i) mosquito biting (49.7%) and (ii) bad or contaminated water (35.1%). In line with the information gathered in the in-depth interviews, it was further inquired whether or not malaria could be caused by drinking water; 71.4% responded affirmatively.

### Local conceptions of malaria.

In the local socio-cultural context, malaria is interpreted according to humoral illness conceptions and classified as a hot disease. The humoral illness theory consists of a classificatory system of interpreting illnesses, categorizing them according to their hot and cold qualities or characteristics.^24^ Both categories, with their internal gradations, do not apply just to illnesses but also to types of treatment (medication and medicinal plants), food and drink, people (i.e., elderly versus children), states of the body (i.e., pregnancy and birth), activities (working versus resting), etc. Illness is the disruption of the natural balance or equilibrium of the humors in the body because of sudden (external) or excessive change, such as cold rain when working hard, hot food when the body is cold or weak, etc. These disrupting actions shock (inf. *chocar* | n. *choque*) or disrupt the health equilibrium that balances the cold and hot humors, leading to weakness and/or illness. Therefore, febrile illnesses such as malaria lead to a state in which the body becomes too hot and during which the patient should avoid an excess of heat (i.e., working in the sun, eating certain hot meals and drinks such as difficult to digest meat like pork, and having sexual relations) or sudden cold (i.e., exposure to rain). However, although malaria is considered a hot disease, it initially undergoes a cold phase during which people experience the characteristic chills or cold fever.

### Local conceptions of treatment.

Regaining good health consists of progressively and slowly restoring the humoral balance and avoiding excess. A hot illness will have to be treated by refreshing the body progressively and will worsen with hot medication and/or with immediate and/or excessively cold medication. However, CQ-PQ treatment is interpreted as having hot properties because of its strength, bitterness (and associated purging effects), and perceived potent effects on the patients' health.

**Figure 2.**
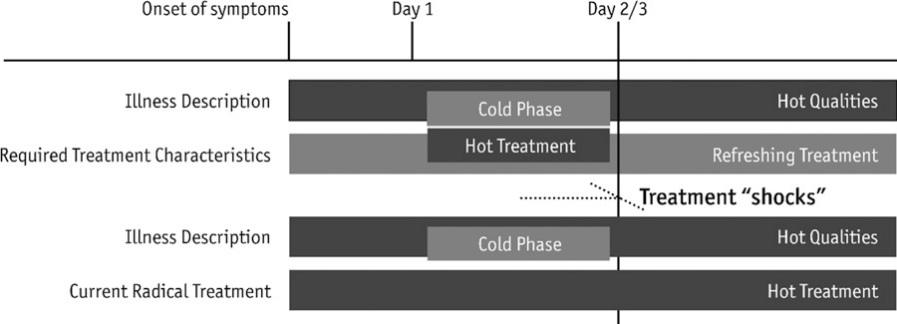
Schematic presentation of humoral illness and treatment conceptions.

Regarding patients' perception of the evolution of their illness in relation to malaria treatment, the following can be highlighted: (i) patients state feeling worse with the first intake of treatment; (ii) almost two-thirds of the patients state feeling better 3 days after initiating treatment (Table 2); (iii) a similar proportion of patients claims that the treatment shocks them within the first 3 days (referring to the disruption of the health equilibrium when the hot state of the body is aggravated by the perceived hot treatment; Table 2); and (iv) the hot properties of malaria lead to the use of refreshing herbal drinks during treatment (75.7% of patients) and the prohibition of hot foods and activities (data not shown). This conception of malaria and its treatment is summarized in Figure 2 and illustrated in the following excerpt from an in-depth interview: “My mother-in-law bought some pork and we went over for lunch but then, the same evening, I was shaking with malaria … My mother-in-law says we're never ever going to buy pork again! … So much malaria with all that pork!” (mestizo farmer; Villa Buen Pastor).

### Delay and treatment before consultation at HC.

According to the Adherence Survey respondents, 58.1% of patients sought biomedical treatment within the first 3 days of symptom onset, whereas an additional 20.7% sought treatment between day 3 and day 6; 21.2% waited for 7 or more days to seek treatment (data not shown). During this pre-medical phase, paracetamol (49.6% of the surveyed population) and some form of traditional treatment, such as bitter medicinal roots and barks, and refreshing herbs (28.3%) were used to reduce fever; 11.8% of respondents stated that one should not to take any medication or consume any special food or drink during this period, and only 2.4% purchased anti-malarial medication outside of the HC (7.9% of responses were missing).

### Productivity time loss.

The median reported number of working days lost because of a clinical vivax attack, among the 185 interviewed patients, was 9 (ranging from 0 to 90 days).

### Adherence to current radical treatment.

#### Doses delivered by the HC.

According to the HCs' clinical files (Table 3), 77.9% (835/1,072) of the vivax patients that consulted at the HC received the full 7-day dose of PQ, and an additional 21.4% received between 3 and 6 days of treatment. Most vivax patients (97.2%) had been given the full 3-day dose of CQ.

#### Doses reportedly taken according to Adherence Survey respondents.

Of all respondents interviewed, 71.9% (133/185) stated that they had taken the treatment for the full 7 days (Table 4). However, when comparing survey response to the HC patient records, only about one-half of the respondents (91/185 = 49.2%) both stated to have completed the 7-day treatment and had actually received the complete 7-day treatment from the HC. Thus, of all respondents that stated to adhere to the full 7-day treatment, about one in three (42/133 = 31.6%) had not returned to the HC to retrieve the medication for the remaining 4 days. Furthermore, 20.2% (23/114) of patients having received the full dose of anti-malarial medication at the HC did not complete the treatment. Considering that 77.9% of the vivax patients consulting at the HC had received the full 7-day dose of PQ (Table 3) and that 20% of them did not complete it, only 62.2% of patients had actually taken the full course of PQ.

### Evaluation of radical treatment.

Perceived difficulties for treatment adherence were evaluated in several survey questions; 69.9% of respondents stated that the medication provided at the HC shocked their health, whereas 61.2% claimed to have had an allergic reaction to the medication. Among respondents that openly admitted to having abandoned treatment (*N* = 37), the same major health-related reasons were cited, especially the fact that the treatment shocked (58.2%); 29.1% did not state any difficulties, whereas minor reasons included allergies (3.6%), having recovered their health before the medication was finished (3.6%), and anorexia (3.6%). All respondents (*N* = 185) were further asked why people generally abandon treatment after 3 days. To this half-open question, major reasons given were related to the medication being bad for people's health (48.7%, including the treatment shocks, allergies, bad for health, makes people weak and pale, etc.) and having recovered good health (10.8%), whereas one in three answered not to know any reasons (30.8%); 9.7% provided other responses.

### Timing of treatment abandonment.

In*-*depth interviews on adherence showed that most non-adherent patients abandoned treatment after the first 3 days. Several reasons can account for this: (i) the progress of the illness and the effect of the medication make patients gradually feel better after 3 days (Table 2); (ii) the perceived shock to the body (Table 2); and (iii) the necessity of patients having to go back to the HC to receive the remaining doses of medication after the first 3 days, which implies new costs (especially transportation costs) and productivity time loss.

### Relapse.

Approximately 24% of patients (45/185) stated to have relapsed after their last vivax malaria episode. Main reasons cited for relapse were eating greasy (hot) food (i.e., pork and chicken; 60.0% = 27/45), drinking alcohol (22.2% = 10/45), an infected mosquito bite (2.2% = 1/45), didn't complete treatment (2.2% = 1/45), and bad water (2.2% = 1/45). Other causes were the rain, using ice, and too much sun (total of 11.1% = 5/45).

### Use of the health promoter.

Twelve percent of all interviewed patients had consulted the health promoter for their last vivax episode, whereas the remaining 88% went directly to the HC or Iquitos hospital.

## Discussion

According to the HCs' records, most patients with vivax malaria had taken a complete treatment with CQ and PQ. Nevertheless, when analyzing such records against survey data, the proportion of patients having taken the full CQ and PQ course was much lower. Response bias was anticipated, because survey respondents generally tend to claim adherence to prescribed public-health interventions, irrespective of their personal experience, opinions, or actions, to avoid being seen as negligent and/or ignorant of basic biomedical health ordinances. Similarly, this holds true for the under-reporting of traditional herbal medicine and other non-biomedical health-seeking practices. These considerations accounted for one of the main reasons for using methodological triangulation.

Most non-adherent patients in the study area abandoned the treatment after or around the initial 3 days. Various factors account for this. Adherence is a continuous process during which the patient evaluates and re-evaluates the course of his illness and the perceived benefits and risks of the treatment. In the local context, treatment of vivax malaria can be seen as consisting of two phases, a first 3-day phase with CQ and PQ and a second 4-day phase with only PQ. During the first phase, patients feel extremely sick and are, therefore, most willing to accept the treatment, despite its perceived side effects. However, during the second phase, as the illness progresses and the effects of the medication take hold, symptoms abate, diminishing the perceived benefits of continuing the remaining 4 days of PQ treatment. Additionally, patients have to return to the HC to collect the remaining PQ tablets. This can be inconvenient in terms of work obligations, costing money, and seeming unnecessary.

Patients believe that the current radical treatment with CQ and PQ, although necessary, is bad for their health. Perceived allergies to the medication and/or other side effects mainly associated with PQ (such as gastrointestinal side effects including nausea, vomiting, stomach pain, etc.) are related to the local humoral illness conception of malaria and its categorization as a hot disease. Malaria, even in its latent form, produces a state of body that is too hot, and as such, the patient should avoid an excess of heat or sudden cold. The consumption of certain excessively hot foods or activities can all exacerbate the malaria sufferer's condition, aggravating symptoms or leading to relapses. In this context, the treatment falls under the category of a product inducing excessive heat in the body. However, despite the fact that malaria is a hot disease, it undergoes an initial cold phase during which the patient develops a cold fever. Vivax malaria symptoms are characterized by febrile peaks of up to 40.5°C, which are preceded, for a half an hour or so, by chills and rigors.^25^ It can, therefore, be hypothesized that a hot treatment is generally more acceptable during the initial stage of the illness where it is perceived to combat the cold. After this phase is over, however, and malaria regains its hot qualities, the medication provided at the HC is considered excessive and consequently unhealthy. Patients, therefore, try to balance the excess heat with drinks and herbs to refresh the body and to counteract the added hot characteristics of the medication. A common course of treatment was to take herbal medication during and after the CQ and PQ treatment. Herbal medication is believed to be healthier than the HC's medicine, is thought to facilitate a faster return to work activities, and, for part of the population, has the added value of being part of traditional culture.

A second example of the humoral illness interpretation of malaria is the local understanding of relapse. Of the about one-quarter of patients believed to have relapsed after the initial *P. vivax* malaria episode, a large majority attributed it to excessive heat or sudden cold, eating greasy food, drinking alcohol, exposure to rain, ice, too much sun, etc. It is in this context that the high percentages of perceived allergies and perceived shock because of the medication have to be understood. Perceived adverse effects typically start at day 2 to day 3, when patients feel a diminished need to continue the treatment. The importance of perceived adverse effects were also a main reason for non-adherence in a study carried out in the province of Esmeraldas in Ecuador, in which adherence was estimated at 65.9% based on direct interviews, including clinically confirmed vivax and falciparum patients.^26^ A qualitative study in Piura and Tumbes in Peru also cited the quick lessening of symptoms and the perceived adverse effects of treatment as the main elements diminishing adherence.^27^

The relatively low adherence to vivax malaria treatment can probably further be related to various general adherence factors, such as the length of the treatment and the inherent milder symptoms of vivax compared with falciparum malaria. However, despite the general assumption that length of treatment and illness severity directly influence adherence, there is limited research and theory in the malaria field to corroborate this.^5^,^28^ The same holds true for distance and access to health care. Despite harsh weather and road conditions during the rainy season, access to the HCs is relatively easy for most communities along the Iquitos/Nauta road, and treatment is free of charge. Furthermore, health staffs suggested that patients in communities with difficult access to treatment might be more willing to adhere out of fear of relapse, whereas in nearby communities, patients often opt to stop treatment as soon as possible because of their easy access to new medication if necessary.

In the present context, several possible policies encouraging greater adherence should be further evaluated. The combination of treatment with certain nutritional or medicinal supplements may increase the perceived supplementary benefits of treatment, especially in a context where the use of supplementary herbal treatment is already a general practice. Increasing community awareness concerning the need to complete PQ intake to avoid further transmission to family and community members could further stimulate social pressure to adhere. Lastly, a feasible system of direct observed intake of the medication, organized at the community level (i.e., by health promoters paid per patient that completes treatment), could be an option, requiring, however, a good supervision system and attractive incentives.

In conclusion, because PQ is the only available drug currently on the market for eliminating *P. vivax* hypnozoites, treatment adherence is essential in light of malaria-elimination strategies and the prevention of parasite resistance. Understanding related illness and treatment conceptions among local populations is, therefore, the key to improving vivax malaria control in endemic regions.

## Figures and Tables

**Table 1:** Perceived causes of malaria among *P. vivax* patients

Answers	Frequency	
Cause of last malaria infection?	*n*	%
Water	65	35.1
Mosquitoes bites	92	49.7
Don't know	22	11.9
Other	6	3.3
Can water cause malaria?		
No	50	27.0
Yes	132	71.4
Don't know	2	1.1
Missing	1	0.5

Total number of interviewed people (*N* = 185).

**Table 2:** Reported time of treatment effects after initiation of PQ treatment

Answers	Frequency	
Reported time people feel better after initiating treatment	*n*	%
0 days	0	0
1 day	8	5.1
2 days	25	15.8
3 days	99	62.7
4 days	25	15.8
6 days	1	0.6
Reported time the treatment starts to shock		
Since the beginning	27	17.1
1 day	55	34.8
2 days	12	12.0
3 days	10	6.3
4 days	5	3.2
5 days	1	0.6
6 days	6	3.8
It didn't shock me	35	22.2

Total number of interviewed people (*N* = 158).

**Table 3:** CQ and PQ doses delivered at the HCs (*N* = 1072)

Treatment delivered	Frequency	
CQ doses delivered	*n*	%
0 day**s**	2	0.2
1 day	3	0.3
2 days	12	1.1
3 days	1.042	97.2
Missing	13	1.2
PQ doses delivered		
< 3 days	10	0.9
3 days	170	15.9
3–7 days	49	4.6
7 days	835	77.9
Missing	8	0.7

**Table 4:** Treatment adherence according to patients' files (HC) and survey respondents

Treatment reportedly taken (survey)	Treatment delivered by HC*		Total
	< 7 days	7 days	*n* (%)
< 7 days	14 (0.2)	23 (20.2)	37 (20.0)
7 days	42 (0.8)	91 (79.8)	133 (71.9)
Missing			15†
Total	56	114	185

* Column percentages shown in parentheses.

† Information missing in the HC files.
